# Anatomical indicators of *Eucalyptus* spp. resistance to *Glycaspis brimblecombei* (Hemiptera: Aphalaridae)

**DOI:** 10.7717/peerj.13346

**Published:** 2022-05-13

**Authors:** Fernando Henrique Moreno de Oliveira Del Piero, Carlos Frederico Wilcken, Maurício Magalhães Domingues, Ana Laura Favoreto, Roberto Antonio Rodella, Alexandre Igor Azevedo Pereira, Wiane Meloni Silva, José Eduardo Serrão, José Cola Zanuncio

**Affiliations:** 1 Departamento de Proteção Vegetal, Faculdade de Ciências Agronômicas, Universidade Estadual Paulista (UNESP), Universidade Estadual Paulista, Botucatu, São Paulo, Brazil; 2 Instituto de Biociências, Universidade Estadual Paulista (UNESP), Universidade Estadual Paulista, Botucatu, São Paulo, Brazil; 3 IF Goiano, Campus Urutaí, Rodovia Geraldo Silva Nascimento, Fazenda Palmital, Instituto Federal Goiano, Urutaí, Goiás, Brazil; 4 Departamento de Engenharia Florestal, Universidade Federal de Viçosa, Universidade Federal de Viçosa, Viçosa, Minas Gerais, Brazil; 5 Departamento de Biologia Geral, Universidade Federal de Viçosa, Universidade Federal de Viçosa, Viçosa, Minas Gerais, Brazil; 6 Departamento de Entomologia/BIOAGRO, Universidade Federal de Viçosa, Universidade Federal de Viçosa, Viçosa, Minas Gerais, Brazil

**Keywords:** Anatomical leaf characterization, Red gum lerp psyllid, Leaf damage

## Abstract

The total area of forest crops in Brazil is 9.55 million hectares, of which 7.5 million hectares are *Eucalyptus*. These crops are the most productive in the world, but may suffer losses due to exotic pests, including *Glycaspis brimblecombei* Moore (Hemiptera: Aphalaridae) found in Brazil since 2003. Interactions between *Eucalyptus* plants and insect pests may led to the selection of resistant genotypes. *Eucalyptus* species are either susceptible or resistant to this pest group, but the damage they suffer needs to be evaluated. The objective was to determine possible leaf anatomy indicators of different *Eucalyptus* species associated with *G. brimblecombei* infestations, focusing on plant resistance to this pest. The study was carried out with *Eucalyptus camaldulensis*, *Eucalyptus grandis*, *Eucalyptus saligna* and *Eucalyptus urophylla* saplings infested or not by *G. brimblecombei* eggs and nymphs. Eighteen anatomical characteristics of the leaves of these plants were analyzed. The number of stomata on the adaxial and abaxial sides and the glandular area in the central leaf vein are associated with greater or lesser infestation by *G. brimblecombei* in the *Eucalyptus* genotypes.

## Introduction

Globally, forest crops cover around 294 million hectares (Food & Agriculture Organization of the United Nations, 2020). Brazil accounts for 9.55 million hectares of this area, with 7.5 million being *Eucalyptus*. Forest plantations in Brazil are among the most productive in the world with 36.8 m³/ha year and with economic, social and environmental importance (Indústria Brasileira de Árvores, 2021). Native and exotic pests can compromise this productivity (Floris et al., 2020; Pereira et al., 2022). *Eucalyptus* plantations are established in large contiguous areas that provide a significant quantity of food and shelter for insect pests (Wingfield et al., 2008).

Exotic pests, introduced in the last two decades, are causing losses to the Brazilian forestry sector (Paine, Steinbauer & Lawson, 2011; Almeida et al., 2018). In 2003, *Glycaspis brimblecombei* Moore (Hemiptera: Aphalaridae) was reported in Brazil (Wilcken et al., 2015) and has reduced crop yields (Saliba et al., 2019). This insect feeds only on *Eucalyptus* species (Wilcken et al., 2015) and leaf rolling and deformation, “witch broom”, dieback and sooty mold are the main features of its infestation (Dittrich-Schroder et al., 2021).

Control methods for *G. brimblecombei* should focus on breeding and planting resistant eucalypt varieties, especially in areas with large *G. brimblecombei* populations (Jere et al., 2020). Different environmental conditions influence host plant susceptibility and infestation levels in the field (Ferreira-Filho et al., 2017; Bush, Slippers & Hurley, 2020).

Leaves, allelochemicals (tannins, phenols and waxes), glands that produce essential oils, often rich in terpenoids, hardness (sclorophilia), heterophilia (differentiation between young and mature leaves) and high regrowth of *Eucalyptus* plants can affect insect damage to this plant, with potential to select for resistant genotypes (Ohmart & Edwards, 1991).

Leaf anatomy is poorly studied and may allow us to understand pest infestations and the development of new tools for their management. Developing integrated psyllid management in *Eucalyptus* plantations depends on knowledge of plant/insect interactions. The objective of this study was to determine possible indicators based on leaf anatomy of four *Eucalyptus* species associated with *G. brimblecombei* infestations. These indicators may be useful in breeding programs for plant resistance to this pest.

## Materials and Methods

The study was carried out at the Universidade Estadual Paulista (FCA/UNESP) in Botucatu, São Paulo state, Brazil. *Eucalyptus camaldulensis*, *E. grandis*, *E. saligna* and *E. urophylla* were planted in 1.5 L pots with an autoclaved mixture of soil: sand: manure (2: 1: 1) and kept in a greenhouse for infestation with *G. brimblecombei*.

The *Eucalyptus* species were previously classified according to their response to *G. brimblecombei* with *E. saligna* and *E. urophylla* being resistant, *E. grandis* tolerant and *E. camaldulensis* susceptible to damage (Brennan et al., 2001; Pereira et al., 2013; Ribeiro et al., 2015).

### Infestation of the *Glycaspis brimblecombei* on *Eucalyptus* plants

*Glycaspis brimblecombei* eggs and nymphs, collected in the field on *Eucalyptus* leaves, were placed on 25 cm high saplings of this plant in the laboratory. Each of the plants was infested with approximately 40 nymphs and two egg masses (more than 25 eggs each), weekly, for 4 weeks.

Twenty seedlings of each *Eucalyptus* species were used per treatment, with 10 plants (replications) infested with *G. brimblecombei* and another 10, as a control, free from the pest. All the plants in the control were sprayed with systemic insecticide (acephate) and the others only with water, to evaluate the effects of mechanical action of the water.

### Anatomical characterization of *Eucalyptus* leaves

*Eucalyptus camaldulensis*, *E. grandis*, *E. saligna* and *E. urophylla* leaves, infested or not, were analyzed. The samples were one to two leaves from the middle third of each eucalypt sapling, cut in three parts with the middle third analyzed. These samples were placed in formaldehyde + glacial acetic acid + 50% alcohol fixative solution (FAA-50) for 48 h and stored in 70% ethanol (Johansen, 1940). The samples were submerged into glyco-methacrylate resin (Gerrits, 1991) and cut, transversely, in a manual microtome, in the internervural region and in the central rib, with 15 to 25 μm thickness. The pieces were cleared, stained with acid fuchsin (Brennan, Weinbaum & Pinney, 2001) and toluidine blue pH 4.7 and mounted in synthetic resin (O’Brien, Feder & Mccully, 1964).

The thickness and the area with the epidermal, parenchymal and vascular leaf tissues were obtained with the computer program Image Tool 3.0 (UTHSCA) to evaluate the damage by *G. brimblecombei* on infested leaves. The quantitative anatomy was performed for three plants (replications) per species of *Eucalyptus* infested or not by *G. brimblecombei*. Eighteen variables for anatomical characterization of the leaf were evaluated.

### Quantitative variables of leaf anatomical characteristics

The 18 variables related to leaf anatomy were: percentages of upper (%UE) and lower (%LE) epidermis, collenchyma (%Col), phloem (%Ph), xylem (%Xy), chlorenchyma (%Chl), gland (%Gl), and total cross-sectional area (mm^2^) (CS) in the region of the central rib, thickness of the upper (TUE) and lower (TLE) epidermis, upper (TUPP) and lower (TLPP) palisade parenchyma, spongy parenchyma (TSP), leaf (TL), mesophyll (TM), the mean area of a gland (MGA), and number of stomata/mm^2^ of the upper (NUS) and lower (NLS) surfaces in the internervure region (Sambugaro et al., 2004).

### Statistical analysis

The anatomical leaf characterization data were subjected to multivariate statistical tests of Cluster Analysis and Principal Component Analysis (PCA) (Sneath & Sokal, 1973) to verify the discriminatory capacity of the quantitative anatomical variables obtained by the measurements of the different leaf tissues, and the means compared by the Tukey test at 5% probability, using R Studio software.

## Results

### Damage by *Glycaspis brimblecombei*

The infestation of *G. brimblecombei* was constant with low plant mortality. *Eucalyptus camaldulensis* was more infested than *E. urophylla* and *E. grandis* and all *G. brimblecombei* nymphs died in the first instars on *E. saligna* without development on plants of this species. Sooty mold developed on *G. brimblecombei* lerps. The occurrence of leaf spot from *Teratosphaeria epicoccoides* was observed on *E. camaldulensis*, *E. grandis* and *E. urophylla* and with greater damage to *E. saligna*.

### Anatomical leaf characterization

The percentage of upper and lower epidermis in the region of the central vein, percentage of collenchyma, thickness of the upper and lower epidermis in the internervure region and thickness of the spongy parenchyma was similar between the *Eucalyptus* species (Table 1). The percentage of chlorenchyma was lowest and that of phloem, xylem and the mean gland area in the central vein region was highest in *E. grandis* leaves than in the other *Eucalyptus* species (Table 1).

**Table 1:** Values of the 18 quantitative anatomical variables for *Eucalyptus camaldulensis*, *Eucalyptus grandis*, *Eucalyptus urophylla* and *Eucalyptus saligna* leaves infested by *Glycaspis brimblecombei* (Hemiptera: Aphalaridae) in a greenhouse.

Variable	*E. camaldulensis*	*E. grandis*	*E. urophylla*	*E. saligna*
Upper epidermis (%)	2.83 ± 0.67a	2.76 ± 1.00a	3.80 ± 0.57a	3.39 ± 0.75a
Lower epidermis (%)	2.42 ± 0.36a	3.00 ± 0.52a	4.11 ± 0.69a	3.90 ± 0.71a
Collenchyma (%)	33.46 ± 10.69a	29.44 ± 4.17a	31.09 ± 4.30a	35.70 ± 6.42a
Phloem (%)	13.90 ± 3.86a	24.74 ± 3.52b	14.97 ± 6.39a	17.41 ± 4.34a
Xylem (%)	16.40 ± 0.52a	19.88 ± 3.61b	12.50 ± 3.78a	10.22 ± 2.46a
Chlorophyll parenchyma (%)	30.12 ± 4.41a	16.03 ± 3.43b	31.36 ± 5.22a	28.11 ± 3.27a
Glands (%)	0.87 ± 0.63a	4.15 ± 1.39c	2.17 ± 2.59b	1.26 ± 1.05b
Total cross-sectional area (mm^2^)	0.61 ± 0.05a	0.57 ± 0.03a	0.31 ± 0.02a	0.46 ± 0.03a
Total of the upper epidermis (μm)	15.94 ± 3.42a	18.44 ± 3.39b	16.56 ± 2.43b	17.19 ± 2.82b
Total of the lower epidermis (μm)	15.31 ± 3.55a	12.19 ± 2.80a	15.31 ± 2.95a	13.75 ± 3.56a
Upper palisade parenchyma	97.19 ± 12.16a	70.94 ± 12.65b	70.00 ± 11.02b	58.12 ± 3.37b
Lower palisade parenchyma	78.44 ± 12.76	0.00 ± 0.00	0.00 ± 0.00	0.00 ± 0.00
Total of spongy parenchyma (μm)	103.75 ± 26.28a	121.25 ± 14.39a	102.81 ± 10.47a	117.19 ± 14.46a
Mesophyll thickness	279.37 ± 77.14a	192.19 ± 59.23b	172.81 ± 32.00b	175.31 ± 48.85b
Leaf thickness (μm)	310.62 ± 52.93a	222.81 ± 50.54b	204.37 ± 23.41b	206.25 ± 58.97b
Mean area of a gland	7.65 ± 2.60a	11.68 ± 2.19a	6.52 ± 0.73a	7.39 ± 0.92a
Number of stomata of the upper surfaces	231.73 ± 20.57a	1.37 ± 0.06b	0.00 ± 0.00	0.00 ± 0.00
Number of stomata of the lower surfaces	256.68 ± 23.89a	500.39 ± 35.71b	527.55 ± 21.01b	557.06 ± 29.43b

**Note:**

Averages followed by the same lowercase letter per line do not differ by Tukey’s test (*p* ≤ 0.05).

**Figure 1:**
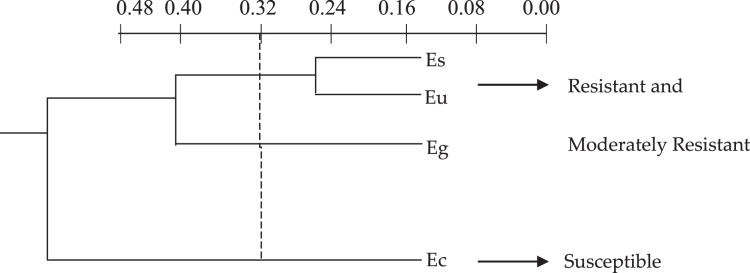
Dendrogram of the cluster analysis of the 18 quantitative anatomical variables of the leaf of four species of *Eucalyptus* infested by *Glycaspis brimblecombei* (Hemiptera: Aphalaridae), using the Average Euclidean Distance. G1: group 1; G2: group 2. Ec: *Eucalyptus camaldulensis*; Es: *Eucalyptus saligna*; Eg: *Eucalyptus grandis* and Eu: *Eucalyptus urophylla*.

The cluster analysis, based on the discriminatory capacity of the quantitative anatomical variables, that is, comparing the elements according to the presence or absence of certain characteristics separated the *Eucalyptus* species into two groups (Fig. 1) based on the low level of 0.32 on the similarity distance scale: group 1–*E. saligna*, *E. urophylla* and *E. grandis*; group 2–*E. camaldulensis*, *E. saligna* and *E. urophylla*.

**Figure 2:**
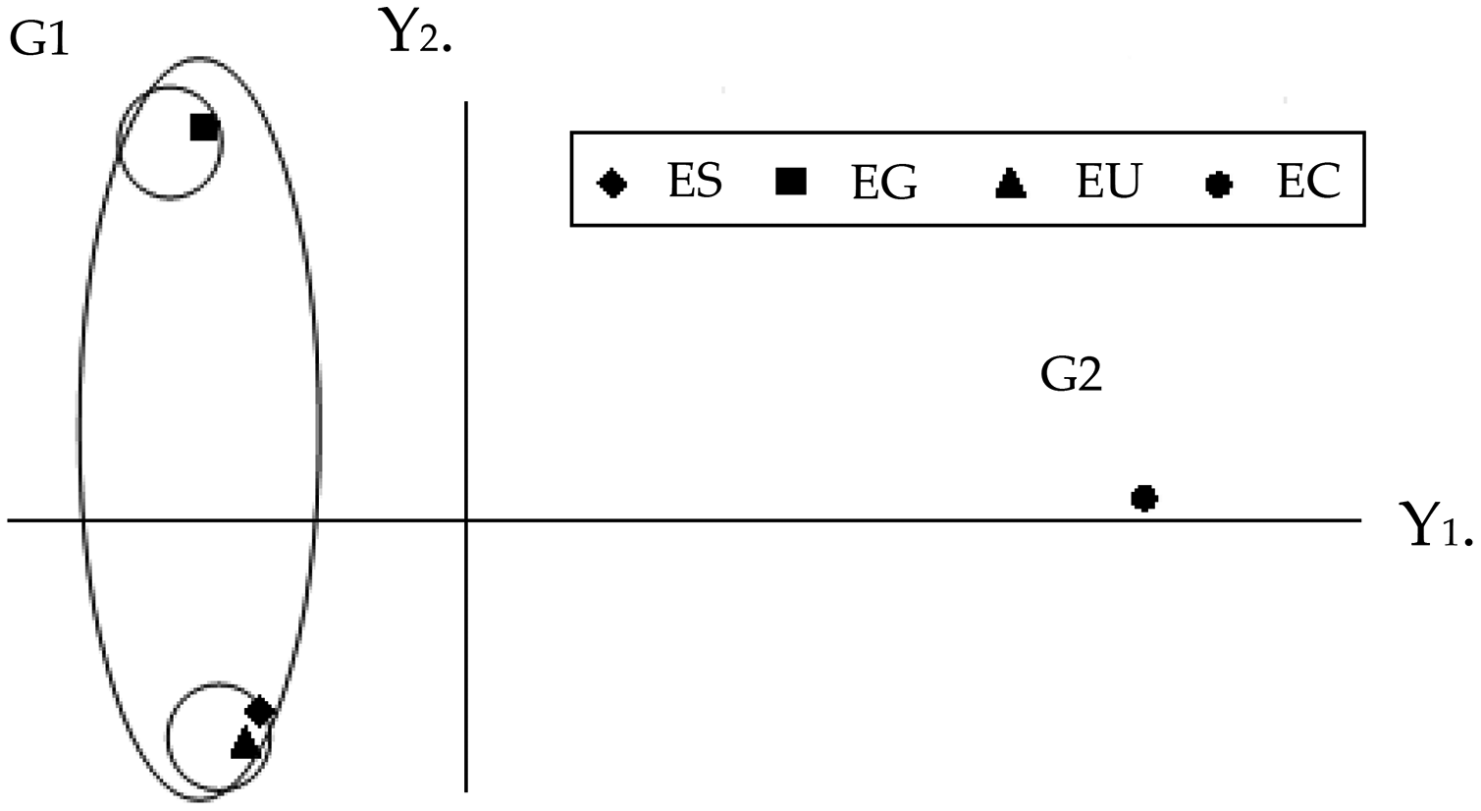
Graphic dispersion of the four species of *Eucalyptus*, using the first two principal components (Y1 and Y2), for the set of 18 quantitative anatomical variables of the leaves infested by *Glycaspis brimblecombei* (Hemiptera: Aphalaridae). G1: group 1; G2: group 2. EC: *Eucalyptus camaldulensis*; ES: *Eucalyptus saligna*; EG: *Eucalyptus grandis* and EU: *Eucalyptus urophylla*.

The graphic dispersion of the four *Eucalyptus* species showed *E. saligna*, *E. urophylla* and *E. grandis* forming group 1 and *E. camaldulensis* group 2 for the principal component analysis with contrast between these species (Y1 and Y2) (Fig. 2). The graphic dispersion of the PCA and the dendrogram of the cluster analysis, grouped the four *Eucalyptus* species into two main groups, based on the 18 quantitative anatomical characteristics of the *Eucalyptus* leaves (Fig. 2).

The correlation coefficients among the 18 quantitative anatomical variables of the *Eucalyptus* leaves and the first two principal components (Y1 and Y2) were found to be thickness variables of the lower palisade parenchyma, mesophyll, leaf, upper palisade parenchyma, upper epidermis, as well as the number of stomata of the upper and lower surfaces. These were the main variables that served to discriminate the four *Eucalyptus* species, based on the first principal component (Y1) (Table 2). The discriminatory power of the absolute value of Y1 for these variables, was high. The information retained for the second principal component (Y2) was low (26.43%), which meant that analysis of this component was unsatisfactory. The combined analysis of the first principal component (Table 2) and the graphic dispersion (Fig. 2) showed that the number of stomata on the lower side, percentage of lower epidermis, thickness of the upper epidermis, and percentage of gland in the central vein of the group 2 species (*E. camaldulensis*) were lower than those of the group 1 species (*E. saligna*, *E. grandis* and *E. urophylla*) (Table 1).

**Table 2:** Correlations between the 18 quantitative anatomical variables retained and accumulated in Y_1_ and Y_2_ for the leaf of *Eucalyptus camaldulensis*, *Eucalyptus grandis*, *Eucalyptus urophylla* and *Eucalyptus saligna* and the first two main components (Y1 and Y2).

Original variables	Y_1_	Y_2_	Original variables	Y_1_	Y_2_
TLPP	0.9987	0.0492	%Ph	−0.5640	0.8062
NUS	0.9984	0.0548	CS	0.5582	0.7198
TL	0.9772	0.2124	TSP	−0.5534	0.5648
NLS	−0.9762	−0.1881	TLE	0.5527	−0.7287
TM	0.9758	0.2186	%UE	−0.4584	−0.8365
TUPP	0.9204	0.2303	%Chl	0.3943	−0.9055
%LE	−0.7627	−0.6427	%Col	0.2953	−0.6390
TUE	−0.7122	0.6655	MGA	−0.2374	0.9642
%Gl	−0.6077	0.7445	%Xy	0.2077	0.9431
%Retained	70.17	26.43	%Accumulated	70.17	96.6

**Note:**

TLPP, lower palisade parenchyma thickness; NUS, number of stomata/mm^2^ of upper face; TL, leaf thickness (μm); NLS, number of stomata/mm^2^ of the lower face in the internervural region; TM, mesophyll thickness; TUPP, upper palisade parenchyma thickness; %LE, Percentage of lower epidermis; TUE, thickness of the upper epidermis; %Gl, gland; %Ph, phloem; CS, total cross-sectional area (mm^2^) in the central rib region; TSP, spongy parenchyma thickness (μm); TLE, lower palisade parenchyma thickness; %UE, percentage of upper epidermis; %Chl, chlorenchyma; %Col, collenchyma; MGA, mean gland area; %Xy, xylem.

**Figure 3:**
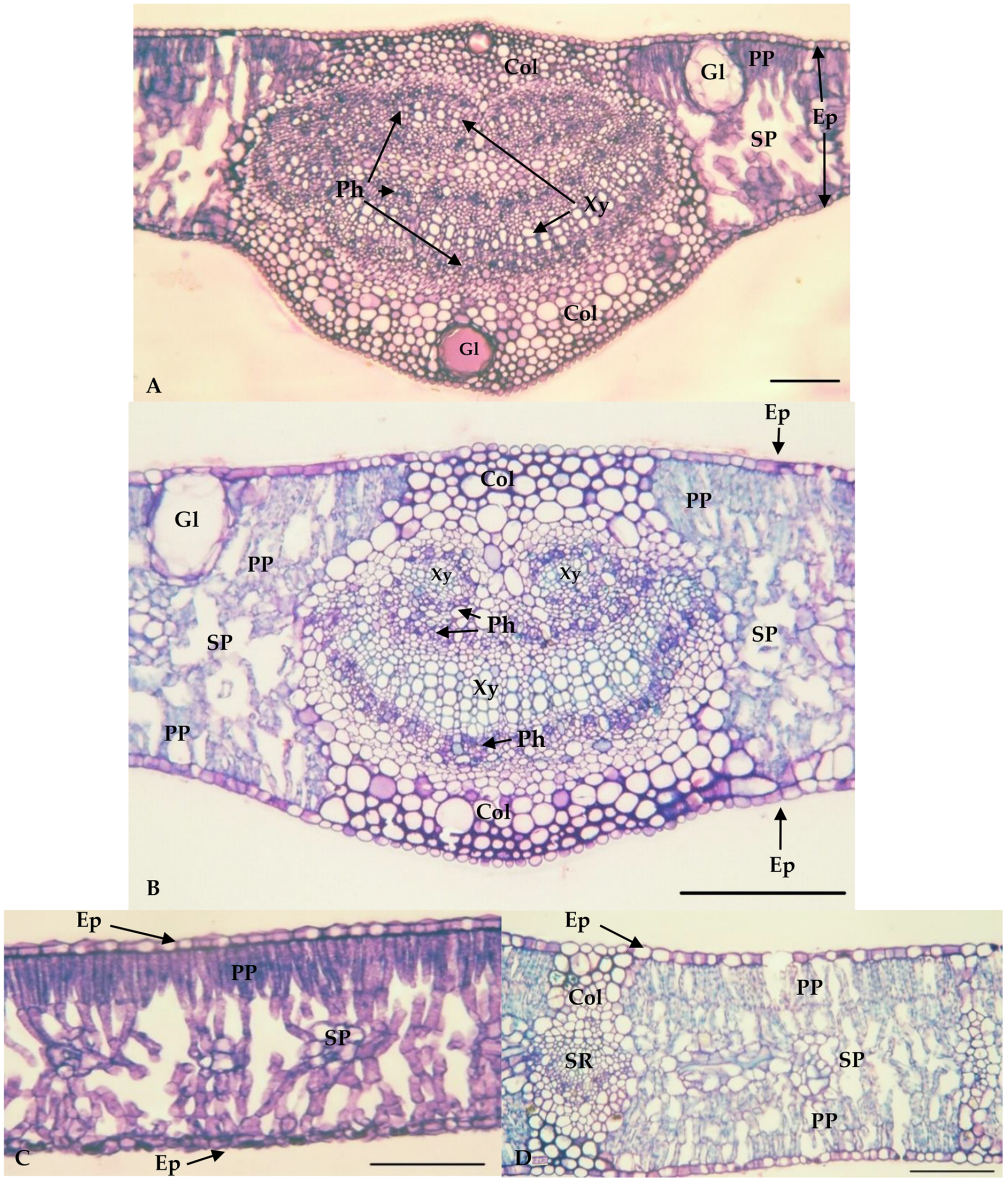
Central vein region of *Eucalyptus grandis* (A) and *Eucalyptus camaldulensis* (B) and internervural of *Eucalyptus grandis* (C) and *Eucalyptus camaldulensis* (D). Bar = 100 μm. Xy = Xylem; Ph = Phloem; Col = Collenchyma; PP = Palisade parenchyma; SP = Spongy Parenchyma; Ep = Epidermis; Gl = Oil gland; SR = Secondary Rib. *Eucalyptus grandis belongs to group 1 (less susceptible); **Eucalyptus camaldulensis belongs to group 2 (susceptible).

**Figure 4:**
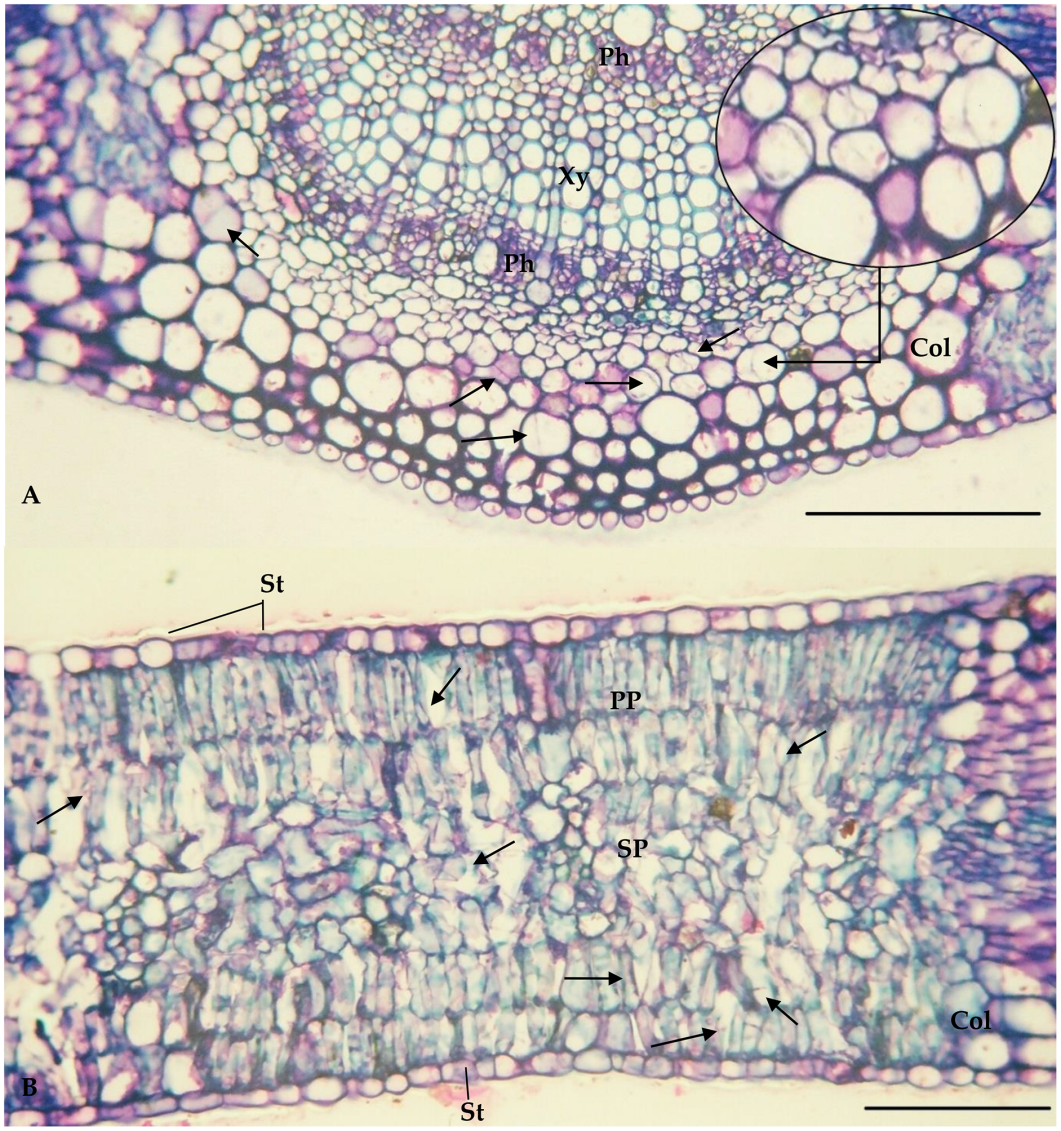
Central vein (A) and internervural (B) region of *Eucalyptus camaldulensis* damaged by *Glycaspis brimblecombei* (Hemiptera: Aphalaridae); Bar = 100 μm Arrow: points where the insect’s stylet passes. Caption: Xy = Xylem; Ph = Phloem; Col = Collenchyma; PP = Palisade parenchyma; SP = Spongy Parenchyma; St = Stomata.

The values of the thickness characteristics of the upper and lower palisade parenchyma, mesophyll and leaf and the number of stomata on the upper surface of *E. camaldulensis* were higher than those for other species. The *E. camaldulensis* leaf profile was classified (Fig. 3). Signs of stylet insertion by *G. brimblecombei* nymphs were found in *E. camaldulensis* leaf sections, passing through the collenchyma, near the central leaf vein and the palisade parenchyma (Fig. 4).

## Discussion

*Glycaspis brimblecombei* damages young plants, from 6 months to mature ones, up to cutting age, causing serious damage throughout its cycle (Saliba et al., 2019). The damage in younger plantations, between 6 months up to 2 years, results in greater losses when compared to more mature plantations (5 years or more) (Wardlaw et al., 2018). *Glycaspis brimblecombei* is a sucking insect and its nymphs produce a large amount of honeydew, causing the development of sooty mold (Reguia & Peris-Felipo, 2013). *Teratosphaeria epicoccoides* on *Eucalyptus* leaves, with greater damage to *E. saligna*, is generally associated with stressed plants (Andjic et al., 2019).

The more intense *G. brimblecombei* infestation on *E. camaldulensis* than on other species tested here is related to its susceptibility to this insect (Firmino-Wincker et al., 2009; Ribeiro et al., 2015). The lack of development of *G. brimblecombei* nymphs on *E. saligna* plants is due to the resistance related to epicuticular wax on the leaves, reducing the presence of eggs and nymphs and the severity of *G. brimblecombei* infestation (Brennan et al., 2001).

The similar percentage of epidermis in the central vein region, collenchyma and epidermis thickness in the internervural region, and thickness of spongy parenchyma for the resistant and susceptible *Eucalyptus* species (Brennan et al., 2001; Pereira et al., 2013; Ribeiro et al., 2015), indicates that these anatomical variables are not associated with the plant resistance or susceptibility to *G. brimblecombei*. The percentage of chlorenchyma, responsible for photosynthesis, is lower in *E. grandis* leaves than in the other *Eucalyptus* species. This is related to a reduction of leaf area, similar to that caused by *Costalimaita ferruginea* (Coleoptera: Chrysomelidae) on shoots and apical parts of *Eucalyptus*, which may reduce chlorenchyma, impairing plant development (Santos, Gonçalves & Silva, 2016). The higher percentage of glands on *E. grandis* leaves in the central vein region, and phloem and xylem in the central vein than in other species may be related to the presence and production of phenolic compounds in the epidermis (Santos et al., 2008), as a result of plant defense to insect pests, including *G. brimblecombei*.

Differences in the number of stomata on the upper surface, and thickness of the upper and lower palisade parenchyma on *E. camaldulensis* due to stomata on the adaxial surface and a double layer of palisade parenchyma on both sides of its leaves. The single layer of palisade parenchyma was found only on the adaxial surface of the other species (James & Bell, 1995).

The palisade parenchyma probably does not confer resistance on *Eucalyptus* spp. to *G. brimblecombei*, because this structure is duplicated on the adaxial and abaxial surfaces of *E. camaldulensis* leaves and single in the adaxial surface of *E. grandis*, *E. saligna* and *E. urophylla*, as well as thicker, on both sides, in *E. camaldulensis* than in the other species. The signs of stylet insertion by *G. brimblecombei* nymphs through the *E. camaldulensis* leaf sections indicates that they passed through the parenchyma cells rather than between them. Cell-degrading proteins such as amylase, cellulase, pectinase and pectinesterase enable stylet entry into the plant tissue (Wu et al., 2021). Stomata are absent or in low numbers in the adaxial surface of *E. grandis*, *E. saligna* and *E. urophylla*, whereas they are present on *E. camaldulensis* leaf side surfaces. The total number of stomata is similar between these species, but this may explain the similar infestation on the abaxial and adaxial surfaces of *E. camaldulensis* compared to *E. urophylla*, with greater infestation on the abaxial surfaces. Stylets of *G. brimblecombei* nymphs penetrated the mesophyll, crossing between the guard cells of the stomata, similar to that observed for this insect in *E. globulus* (Brennan & Weinbaum, 2001a, 2001b) and, for this reason, stomata on both sides of *E. camaldulensis* may confer greater susceptibility to *G. brimblecombei*.

Defense strategies of *Eucalyptus* trees for insects include physical barriers and constitutive and inducible chemical defenses (Patton et al., 2017). The concentration and variability of terpenes, the presence of specific compounds (Silveira et al., 2021), amounts of epicuticular wax in the leaves, and the occurrence of antibiosis, related to longer insect development stages or life cycles, and/or antixenosis resistance, related to extended developmental stages due to lower food intake of insects, are characteristics normally associated with *Eucalyptus* resistance to *G. brimblecombei* (Pereira et al., 2020).

The proportional area and number of stomata occupying the epidermis may also be important for *G. brimblecombei* nymph infestation and to explain *E. camaldulensis* susceptibility to this pest. The thinner epidermis of the adaxial surface and lower percentage of epidermal tissue on the abaxial surface of *E. camaldulensis* leaves are possibly related to the higher susceptibility to *G. brimblecombei*. This is a pioneering study evaluating anatomical foliar indicators in relation to *Eucalyptus* pests, and allows us to better understand pest infestation patterns, and concomitantly, the morphological characteristics that normally confer resistance, such as waxy coating, trichoids, and stomata in these plants.

## Conclusions

The number of stomata in the adaxial and abaxial leaf surfaces and percentage of gland area in the central vein of the leaves are related to the resistance or susceptibility of *Eucalyptus* plants to *G. brimblecombei*.

*Eucalyptus grandis*, *E. urophylla* and *E. saligna*, with higher values of the leaf characteristics evaluated, may be considered resistant or moderately resistant to *G. brimblecombei*.

## Supplemental Information

10.7717/peerj.13346/supp-1 Supplemental Information 1 The raw measurements. The evaluations of the quantitative anatomical variables of the different Eucalyptus genotypes.

## Additional Information and Declarations

### Data Availability

The following information was supplied regarding data availability: The raw measurements are available in the Supplemental Files.
